# Development and Validation of a Kit to Measure Drink Antioxidant Capacity Using a Novel Colorimeter

**DOI:** 10.3390/molecules21091154

**Published:** 2016-08-30

**Authors:** Alexandros Priftis, Dimitrios Stagos, Nikolaos Tzioumakis, Konstantinos Konstantinopoulos, Anastasia Patouna, Georgios E. Papadopoulos, Aristides Tsatsakis, Dimitrios Kouretas

**Affiliations:** 1 Department of Biochemistry and Biotechnology, University of Thessaly, Larissa 41221, Greece; alexandros.priftis@hotmail.com (A.P.); stagkos@uth.gr (D.S.); geopap@bio.uth.gr (G.E.P.); 2 Polytech S.A., Larissa 41222, Greece; nikos.tzioumakis@outlook.com; 3 Coffee Island S.A., Patras 26334, Greece; kostas@coffeeisland.gr; 4 Department of Agricultural Engineering Technologists, TEI of Thessaly, Larissa 41110, Greece; anastasia.pat93@hotmail.com; 5 Department of Forensic Sciences and Toxicology, Medical School, University of Crete, Heraklion 71003, Greece; aris@med.uoc.gr

**Keywords:** kit, antioxidant, quality control, DPPH, colorimeter, drink, beverage

## Abstract

Measuring the antioxidant capacity of foods is essential, as a means of quality control to ensure that the final product reaching the consumer will be of high standards. Despite the already existing assays with which the antioxidant activity is estimated, new, faster and low cost methods are always sought. Therefore, we have developed a novel colorimeter and combined it with a slightly modified DPPH assay, thus creating a kit that can assess the antioxidant capacity of liquids (e.g., different types of coffee, beer, wine, juices) in a quite fast and low cost manner. The accuracy of the colorimeter was ensured by comparing it to a fully validated Hitachi U-1900 spectrophotometer, and a coefficient was calculated to eliminate the observed differences. In addition, a new, user friendly software was developed, in order to render the procedure as easy as possible, while allowing a central monitoring of the obtained results. Overall, a novel kit was developed, with which the antioxidant activity of liquids can be measured, firstly to ensure their quality and secondly to assess the amount of antioxidants consumed with the respective food.

## 1. Introduction

All aerobic organisms are subject to damage from reactive oxygen species (ROS) [1]. ROS are mainly produced by the aerobic metabolism due to the imperfect reduction of O_2_ to H_2_O, as 1%–5% of oxygen gets reduced to the superoxide anion (O_2_^●−^), from which, in turn, the hydroxyl radical (HO^●^) occurs [2]. However, other sources of ROS production do exist and may lead to the accumulation of an excessive amount of such species. These sources include the “oxidative burst” of the immune system, the activity of many redox enzymes, as well as environmental factors like pollution or smoking [3]. ROS when in excess may cause severe damage to cellular macromolecules, a condition known as oxidative stress that has been associated with many diseases [4,5,6]. Living organisms have developed a delicate arsenal of endogenous antioxidant mechanisms to keep at a minimum the harmful effects of free radicals, however, sometimes these mechanisms do not suffice [4,7]. Therefore exogenous supplementation of antioxidants is considered necessary, mainly through food (either liquid or solid) [8,9]. Antioxidant foods have been the focus of numerous studies, in the endeavor to elucidate their properties and how they could be used to improve the overall antioxidant defense system of the human organism [8,9,10]. Attention has been mainly drawn towards polyphenols that constitute an important part of both edible and non-edible plant parts. Polyphenols are the most abundant plant secondary metabolism products (their number exceeds 8000 species) implicated in many characteristics of plants like color and defense [11]. Many studies have been conducted on polyphenols as they display strong antioxidant properties, among other biological activities [12].

For example, mitochondrial homeostasis may be altered by plant bioactive compounds like resveratrol through the modulation of the AMPK and TOR signaling pathways, favoring mitochondrial biogenesis and mitophagy, a process that eliminates old and malfunctioning mitochondria from the cell thus diminishing excess ROS production. In addition, such compounds may affect the mitochondrial-related apoptosis pathway [13,14]. 

Therefore, consuming foods and drinks derived from plants may provide the human organism with useful antioxidant molecules that can be employed to improve redox status. However, each plant contains different antioxidants at various amounts, leading to profound differences in the antioxidant capacity of foods and drinks originating from the processing of plants [15]. Therefore, being able to calculate each food's antioxidant capacity would provide useful information, and for this reason food industries conduct such checks routinely, mainly by using the Oxygen Radical Absorbance Capacity (ORAC) assay [16,17]. However, the equipment and the assays they use are quite expensive [18]. As a consequence, new fast, non-expensive assays and the respective equipment are sought, so that not only food industries, but other organizations or professionals will be able to perform food quality checks. In an effort to achieve this aim, a novel colorimeter with an accompanying software were developed as a kit that employs a slightly modified 2,2-diphenyl-1-picrylhydrazyl (DPPH) assay to estimate the antioxidant capacity of commonly consumed drinks such as coffee, wine and juices and beer. The DPPH assay relies on the scavenging of this artificial mauve radical. Generally there are two ways to neutralize a free radical, namely Single-Electron Transfer (SET) and Hydrogen Atom Transfer (HAT) and DPPH can be neutralized by both from antioxidant compounds, yielding the respective yellow hydrazine [19].

The whole idea for the development of this kit was conceived to provide a means to easily measure the antioxidant capacity of drinks and determine the daily intake of antioxidants.

## 2. Results and Discussion

**Figure 1:**
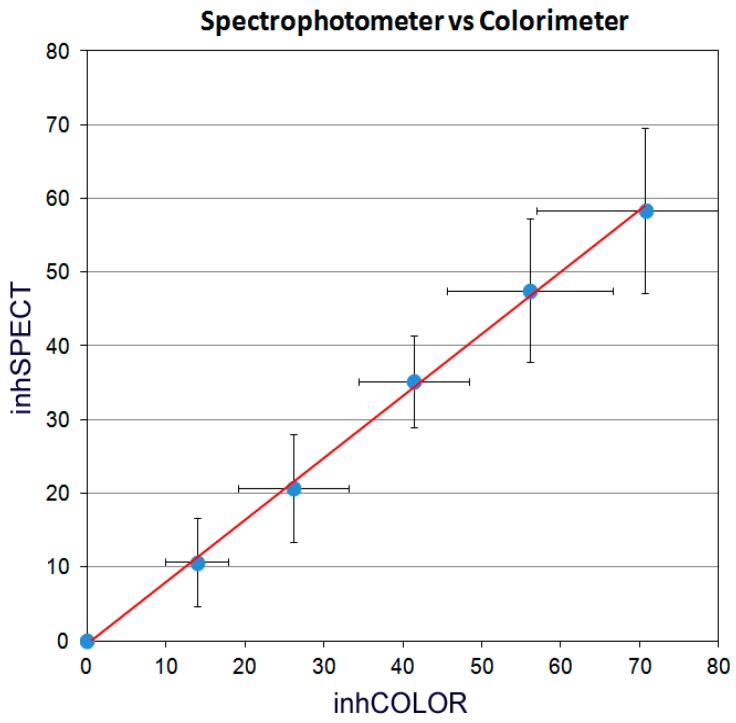
Comparison between the colorimeter and the Hitachi U-1900 spectrophotometer. The graph depicts the correlation between the observed RSC% (or inhibition, inh) from either the colorimeter (inhCOLOR, *x* axis) or the spectrophotometer (inhSPECT, *y* axis). The inhibition levels for each quantity are taken as averages over 17 samples measured three times each (Table 1). The error bars represent SD. The resulting equation of the regression line is inhSPECT% = inhCOLOR% X 0.8417−0.4789 and the R^2^ measure is 0.9988.

According to the 17 tested samples (espresso coffee, biological replicates) which are shown in Table 1, the newly developed colorimeter yielded very similar results compared to the Hitachi U-1900 which was used as a standard. The intraday standard deviation (SD) of the Radical Scavenging Capacity (RSC) was 1.96% for *n* = 5 (one sample measured five times within a day, technical replicates), while the 3-day SD of the RSC for *n* = 5 was 3.80% (five samples measured once daily for three consecutive days). However the 2-day SD of the RSC for the same samples was only 1.94%. This difference can be attributed to a deterioration of the sample's activity. The particular samples were espresso coffee, the activity of which has been found to deteriorate in a time dependent manner in previous measurements in our lab, especially after 24 h (data not published). Therefore the increase in the SD on the third day was expected. Apart from the SD, the Coefficient of Variation (CV) for the same samples was 0.05 for the intraday measurements and 0.09 for the 3-day measurements. In addition, the detection range was from 0.05 μL to 0.70 μL for espresso coffee, and so the limit of detection (LOD) for espresso coffee is 0.05 μL. Furthermore, the assay was very accurate for different concentrations, as 0.2, 0.3 and 0.4 μL of espresso coffee (*n* = 8) yielded potency values of 98.97, 101.52 and 98.48 μmol DPPH scavenged per mL liquid respectively. According to the statistical analysis, the comparison between the colorimeter and the Hitachi U-1900 spectrophotometer (i.e., the reference instrument) showed that the RSC value obtained by the colorimeter can be reliably predicted using the resulting equation (Figure 1).

The regression line was obtained by using the average and SD from all the measurements of the 17 samples shown in Table 1. The difference between the two instruments is linear, however the gap is smaller when low quantity is used and it increases with higher quantity. Thus, it is reasonable to suggest that low quantity is to be used to achieve the highest accuracy.

The Pearson correlation coefficient between the two instruments was 0.999 with *p* < 0.001 (2-sided), while the F statistic was 3199.666 with *p* < 0.001, meaning that the curves obtained from both instruments were practically equal. Finally, it is estimated that an analyst may conduct approximately 30 analyses per hour (including the 20 min incubation).

The developed software, apart from the automation in sample analysis, offers a user-friendly environment that allows for quick measurements while the data from all samples are saved and may be send to a supervised central database (under development), enabling real-time quality control of the currently tested samples.

The DPPH assay was chosen as it is quite fast and cheap, with easily reproducible results and only requires a spectrophotometer (or a colorimeter as shown in the current study), while the protocol is fairly easy and robust and can be performed by non-specialists after a brief introduction. This assay combines the two main mechanisms through which antioxidants act, namely hydrogen atom transfer (HAT) and single electron transfer (SET), therefore it may provide an insight on the overall antioxidant activity of a tested liquid [18].

Table 2 sums up all the liquids that were tested using the colorimeter. This table -as well as the future ones that may be created by using the proposed kit- contains useful information that could be extracted and used to improve one's nutrition according to their needs.

For example, pomegranate juice is the most potent antioxidant source according to this table. A brief examination of the literature shows that pomegranate juice may improve the antioxidant defense system in humans [20]. More specifically, consumption of 500 mL of pomegranate juice for 15 days improved Glutathione (GSH) levels, while a decrease in malondialdehyde (MDA, a lipid peroxidation marker) and protein carbonyls (CARB, a protein oxidation marker) levels was observed. According to this, it could be rendered feasible to calculate the amount of antioxidants required per respective food, in order to achieve a biologically significant result like redox status improvement. According to the table, a dose of pomegranate juice (200 mL) has a potency of 3860 up to 14,280 Units (where 1 Unit is 1 μmol of scavenged DPPH per mL of drink) with a mean of 9070 Units. Therefore, according to the aforementioned study, a total of approximately 22,500 Units (corresponding to 500 mL of pomegranate juice) may yield a useful biological response in the form of increased GSH and lowered oxidative stress. However, as the pomegranate juice is the most potent tested liquid in terms of total Units per dose, in order to achieve that sum of Units higher amounts of the other drinks need to be consumed. Specifically, 2.5 cups of instant coffee which is the second most potent beverage will yield the same amount of Units. The other liquids have even lower potency. For example, 6.7 glasses of juice with pomegranate and other fruits (grape, orange and carrot) or 8.5 cups of espresso coffee are required to reach the required amount of Units. As far as other commonly consumed drinks are concerned, 24.1 glasses of 9 fruit juice and an impressive 8152 glasses of orange or 18145 glasses of peach juice will yield the sought amount. In general, pomegranate juice possesses 2.7 to 9115-fold the antioxidant activity of other juices, 1.01 to 8.98-fold that of various coffees and 30.2 to 110-fold that of the blonde beer, white wine and milks tested. Of course it needs to be taken into consideration that the doses for each respective liquid vary from 30 to 330 mL. This provides an insight on the amount of antioxidants obtained by each of the aforementioned liquids. Nevertheless, the subjects of antioxidant quality over quantity and bioavailability arise, but these are not to be examined on the current study.

Furthermore, the National Institute of Health (NIH) of the Unites States has announced recently the “Precision Medicine” initiative program (www.nih.gov/precision-medicine-initiative-cohort-program). This initiative aims on elucidating the underlying mechanisms of many pathological conditions taking into consideration individual genetic profile differences, so as to produce the best course of action to prevent or cure these conditions for each individual separately. Up until now, the conventional therapies utilized by doctors were useful for the majority of the population, but not for everyone as there were always cases of ineffective treatments. This applies not only for drugs but also for diets, as there is no single ‘golden standard’ diet with which everyone will have a balanced intake of all required nutrients and other molecules to fit his individual needs. The presented kit may help address this issue by providing an estimation of the consumed antioxidants that may lead to the improvement of one’s redox status.

Overall, a new kit with which the antioxidant capacity of liquids can be readily and reliably examined was developed, allowing for cheap and fast assessment, not requiring delicate equipment and much expertise. The development of another kit that will measure the total antioxidant capacity of humans using a single drop of blood is also underway. The results from these two measurements could possibly be combined to yield a picture of the current redox status of an individual as well as the dietary adjustment that should be performed in order to improve the redox status.

## 3. Experimental Section

### 3.1. Tested Drinks

The various tested coffees, juices, beers and wines were commercial. All drink types were tested three times, with the exception of espresso coffee for which 17 samples were used to validate the colorimeter and produce Equation (3). Five concentrations for each espresso coffee sample were used to validate the colorimeter, namely 0.1, 0.2, 0.3 0.4 and 0.5 μL of coffee per 1000 μL of the reaction volume. For the final protocol, the 0.3 μL concentration was chosen.

### 3.2. DPPH Radical Scavenging Assay

Free-radical scavenging capacity of the extract was evaluated using the DPPH radical [19]. Briefly, a 1.0 mL freshly made methanolic solution of DPPH radical (100 μΜ, Sigma-Aldrich, St. Louis, MO, USA) was mixed with tested extract solution at different concentrations. The contents were vigorously mixed, incubated at room temperature in the dark for 20 min, and the absorbance was measured at the green color of the colorimeter (515–530 nm). In each experiment, methanol alone was used as blank and DPPH alone in methanol was used as control. 

### 3.3. Radical Scavenging Capacity (RSC) and Drink Potency Determination

Radical Scavenging Capacity and drink potency determination are determined automatically from the developed software. The percentage of RSC of the tested extracts was calculated according to the following equation:

RSC (%) = [(A_control_ − A_sample_)/A_control_] × 100 (1)

where A_control_ and A_sample_ are the absorbance values of the control and the test sample respectively.

The antioxidant capacity of each liquid was calculated according to the following equation:

μmol DPPH scavenged/mL liquid = RSC (%) × 0.1 × D
(2)

where RSC (%) is actually the percentage of the μmoles scavenged by the tested liquid; 0.1 is the μmoles of the DPPH in the reaction solution; ‘D’, stands for the dilution of the sample in the solution. In addition, 1 μmol of scavenged DPPH per mL of drink is considered as 1 Unit of activity. All experiments were carried out in triplicate and at least on two separate occasions. The ‘D’ value was calculated for each sample category separately, as different drinks displayed a broad range of potency, therefore inter-category differences had to be taken into consideration. The dilution factor was 3333 for pomegranate juice (30 μL of 1:100 diluted sample were used in the reaction of a total volume of 1000 μL), espresso and instant coffee, 500 for Greek and filtered coffee (1:15), 333.3 for red wine and juices with pomegranate mixed with other fruits (1:10), 100 for juices without pomegranate (1:3) and 33.33 for beer and white wine (no dilution).

### 3.4. Colorimeter Development

The colorimeter and the software were developed by Polytech (Polytech S.A., Larissa, Greece). The developed colorimeter can measure the transmittance of a liquid using a green LED light, covering the wavelength between 515 and 530 nm. It has a transmittance measuring range of 0%–100% with ±0.2% accuracy. Apart from the green light (515–530 nm), it also has a red one (630 nm), a blue one (435–450 nm) and a violet one (390–410 nm).

The transmittance is then transformed to absorbance and consequently to the respective RSC value by the software, which in turn gets normalized according to the following equation that was deducted from the linear curve of Figure 1:

RSC_norm_(%) = RSC(%) × 0.8417 − 0.4789 (3)

Consequently, the RSC_norm_ is automatically converted to the potency value of the tested drink according to Equation (2). In addition, the potency per dose of drink is also calculated to give a better perspective of the amount of antioxidants consumed per drink.

### 3.5. Statistical Analysis

The statistical analysis was performed using the IBM SPSS version 20 software (SPSS, Inc., Chicago, IL, USA). Specifically, the Pearson correlation between the RSCs measured by the colorimeter and the spectrophotometer was calculated, as well as the F statistic and the equation that normalizes the RSC measured by the colorimeter.

## Figures and Tables

**Table 1:** Comparison of the DPPH assay Radical Scavenging Capacity (RSC) obtained from the colorimeter (inhCOLOR) and the Hitachi U-1900 spectrophotometer (inhSPECT). All measurements are the mean ± SD from each triplicate.

Sample	inhSPECT						inhCOLOR					
	0.00 μL	0.10 μL	0.20 μL	0.30 μL	0.40 μL	0.50 μL	0.00 μL	0.10 μL	0.20 μL	0.30 μL	0.40 μL	0.50 μL
1	0.00	18.28 ± 0.52	28.43 ± 0.90	41.30 ± 1.35	59.46 ± 1.96	67.85 ± 2.14	0.00	14.92 ± 0.16	28.62 ± 0.70	47.23 ± 1.35	68.08 ± 2.01	86.75 ± 2.43
2	0.00	7.07 ± 0.16	18.58 ± 0.76	35.62 ± 1.54	48.58 ± 1.55	58.85 ± 1.86	0.00	11.43 ± 0.11	25.39 ± 0.71	40.61 ± 1.15	54.65 ± 1.56	71.64 ± 1.78
3	0.00	8.07 ± 0.24	26.62 ± 1.02	42.30 ± 1.22	55.38 ± 1.33	70.42 ± 1.99	0.00	13.29 ± 0.09	33.57 ± 0.95	51.28 ± 1.44	69.65 ± 2.15	92.59 ± 2.56
4	0.00	17.22 ± 0.51	32.08 ± 1.28	42.90 ± 1.15	55.44 ± 1.24	64.83 ± 2.12	0.00	19.77 ± 0.30	37.09 ± 1.01	51.53 ± 1.65	69.02 ± 2.08	83.24 ± 1.97
5	0.00	18.43 ± 0.35	29.13 ± 1.05	34.79 ± 1.00	50.32 ± 1.01	69.21 ± 2.20	0.00	19.28 ± 0.23	33.32 ± 0.78	40.32 ± 1.04	57.88 ± 1.76	76.63 ± 1.99
6	0.00	18.52 ± 0.43	30.61 ± 0.78	41.65 ± 1.76	53.12 ± 1.86	62.66 ± 1.66	0.00	19.90 ± 0.17	33.90 ± 0.55	50.44 ± 1.47	64.13 ± 1.43	75.21 ± 2.01
7	0.00	7.16 ± 0.12	16.58 ± 0.54	26.38 ± 0.95	32.68 ± 0.97	40.80 ± 0.98	0.00	7.29 ± 0.07	17.47 ± 0.16	28.00 ± 0.67	39.52 ± 1.02	50.17 ± 1.43
8	0.00	6.24 ± 0.09	18.91 ± 0.43	28.80 ± 0.78	30.23 ± 0.78	39.00 ± 0.77	0.00	12.06 ± 0.10	23.40 ± 0.46	32.67 ± 0.88	42.77 ± 1.13	51.81 ± 1.32
9	0.00	6.29 ± 0.11	16.86 ± 0.87	35.23 ± 0.96	45.69 ± 1.06	61.82 ± 2.03	0.00	15.93 ± 0.33	30.33 ± 1.01	44.31 ± 1.21	58.65 ± 1.43	78.37 ± 1.93
10	0.00	16.59 ± 0.39	17.36 ± 0.66	34.48 ± 0.66	43.05 ± 1.15	53.05 ± 1.55	0.00	17.24 ± 0.18	29.80 ± 0.88	42.84 ± 1.00	56.58 ± 1.72	69.10 ± 1.77
11	0.00	9.86 ± 0.20	17.14 ± 0.34	27.37 ± 0.74	36.56 ± 1.05	48.38 ± 1.13	0.00	15.78 ± 0.40	26.30 ± 0.76	38.96 ± 0.73	48.66 ± 1.13	63.75 ± 1.04
12	0.00	0.54 ± 0.01	14.70 ± 0.66	32.70 ± 0.76	44.03 ± 1.24	47.53 ± 1.22	0.00	6.26 ± 0.15	18.28 ± 0.45	34.22 ± 0.89	43.86 ± 1.10	49.90 ± 0.55
13	0.00	3.79 ± 0.09	9.79 ± 0.23	23.58 ± 0.69	36.29 ± 0.99	48.95 ± 1.65	0.00	9.06 ± 0.23	16.38 ± 0.35	32.05 ± 0.80	40.29 ± 1.01	51.51 ± 1.43
14	0.00	8.99 ± 0.12	15.93 ± 0.49	41.25 ± 1.23	60.33 ± 2.01	72.75 ± 2.23	0.00	12.23 ± 0.22	22.70 ± 0.57	42.55 ± 1.10	62.34 ± 1.49	77.31 ± 2.04
15	0.00	11.87 ± 0.25	22.59 ± 0.75	38.70 ± 1.04	55.98 ± 1.96	70.29 ± 2.10	0.00	13.35 ± 0.38	27.61 ± 0.33	41.37 ± 1.14	61.03 ± 1.86	74.01 ± 1.67
16	0.00	4.17 ± 0.07	8.04 ± 0.32	30.48 ± 0.77	41.20 ± 1.29	47.43 ± 1.21	0.00	14.08 ± 0.41	11.86 ± 0.30	38.27 ± 0.99	48.26 ± 1.35	63.16 ± 1.59
17	0.00	18.28 ± 0.67	28.43 ± 1.03	41.30 ± 0.55	59.46 ± 1.78	67.85 ± 1.97	0.00	14.92 ± 0.09	28.62 ± 0.18	47.23 ± 1.21	68.08 ± 1.56	86.75 ± 2.32
Average	0.00	10.67	20.69	35.22	47.52	58.33	0.00	13.93	26.15	41.40	56.09	70.70
SD		6.04	7.32	6.22	9.77	11.19		4.02	7.02	6.96	10.45	13.71

**Table 2:** The summary of the tested drinks.

Drink	Type	Antioxidant Activity (μmol DPPH/mL)	Antioxidant Activity Per Dose ^a^
Pomegranate Juice	−	19.30–71.40	3860–14280
Juice with pomegranate and other fruits (grape, orange and carrot)	−	11.90–21.70	2380–4340
Juice without pomegranate	Orange	1.12–4.40	224–880
	Lemon	0.91–1.08	182–216
	Mandarin	0.82–1.28	164–256
	Apple-Orange	2.27–3.06	454–612
	Apple-Orange-Carrot	1.83–2.75	366–550
	Cranberry-Raspberry-Blueberry	4.36–4.87	872–974
	Sour Cherry	2.56–5.45	512–1090
	Peach	0.72–1.76	144–352
	Blackcurrant	7.11–8.37	1422–1674
	Orange-Apple-Apricot	2.48–3.64	496–728
	Orange-Apple-Carrot	2.34–4.35	468–870
	9 fruits	3.30–6.01	660–1202
Coffee	Espresso Coffee	60.30–116	1807–3489
	Instant Coffee	28.30–43.10	7062–10775
	Greek Coffee	16.40–28.60	735–1285
	Filtered Coffee	5.25–6.35	1522–1841
Milk	Milk (full fat)	0.33–0.49	66–98
Wine	White Wine	0.50–2.50	100–500
Beer	Blonde Beer	1.20	396

^a^ (200 mL for juices, 30 mL Espresso, 45 mL Greek Coffee, 250 mL Instant Coffee, 300 mL Filtered Coffee, 200 mL per glass of milk and wine, 330 mL for beer).
